# The complete chloroplast genome sequence of *Nymphoides peltata* (S.G.Gmel.) Kuntze

**DOI:** 10.1080/23802359.2022.2098851

**Published:** 2022-07-29

**Authors:** Xiangqin Zhou, Yudie Lv, Jingtao Zhang, Dongling Wang, Zihao Wang, Yuan Zhou, Wenfeng Wang

**Affiliations:** College of Tropical Crops, Hainan Key Laboratory for Sustainable Utilization of Tropical Bioresource, Hainan University, Haikou, People’s Republic of China

**Keywords:** *Nymphoides peltata*, chloroplast genome, phylogenomic analysis

## Abstract

*Nymphoides peltata* is an aquatic floating weed widely distributed in the northern hemisphere of Eurasia. To better determine its phylogenetic relationships with other *Nymphoides* species and other aquatic plant species, the chloroplast genome of *N. peltata* was sequenced. The chloroplast genome size is 152,198 bp, consisting of a large single-copy region (84,223 bp) and a small single-copy region (17,817 bp) separated by a pair of inverted repeats with a length of 25,079 bp. The chloroplast genome contains 127 genes, including 85 protein-coding genes, 34 tRNA genes, and eight rRNAs. The maximum-likelihood phylogenetic tree showed that *N. peltata* is more closely related to other *Nymphoides* species, providing new insight into the evolution and genetic diversity of aquatic weeds.

*Nymphoides peltata* (S.G.Gmel.) Kuntze (Revis. Gen. Pl. 1891) is an aquatic floating weed which belongs to the family *Menyanthaceae*. *N. peltata* is widely distributed in temperate and subtropical regions of the northern hemisphere (Ho and Ornduff 1995). The weed lives in ponds, lakes, and slow-moving streams; and it usually roots in the bottom mud and maintains its leaves afloat on the water surface. Therefore, it has been used in experimental model systems to investigate plant responses to heavy metals (Lavid et al. 2001) and flooding stress (Yu and Yu 2011; Wu et al. 2017). To better protect and understand the phylogeny of *N. peltata*, the chloroplast genome has been studied based on high-throughput sequencing approaches.

The wild sample of *N. peltata* was gathered from the Baisha, Haikou, Hainan province (19.15°N, 108.49°E). Collection and research of plant material were carried out by guidelines provided by Hainan University Ethical Committee and comply with local legislation. The voucher specimen (Np-WWF-20210625) was deposited at Hainan Key Laboratory for Sustainable Utilization of Tropical Bioresource (Wenfeng Wang, 993906@hainanu.edu.cn). Total genomic DNA was extracted from leaves and sequenced through Illumina NovaSeq 6000 by the Beijing Novogene Bioinformatics Technology Co., Ltd. (Beijing, China). The whole chloroplast genome DNA was assembled by the NOVOPlasty v4.2 (Dierckxsens et al. 2020). The genome was annotated by the GeSeq using the parameters as follows: Protein search identity: 60; rRNA, tRNA, DNA search identity: 35 (Tillich et al. 2017). Maximum-likelihood analyses were performed based on the GTR + G+I model with 1000 bootstraps by MEGA X (Kumar et al. 2018).

The complete chloroplast genome of *N. peltata* is a circular molecule of 152,198 bp in length with base compositions of 31.0% A, 19.0% C, 18.2% G, and 31.8% T. The genome is consisting of a large single-copy region (LSC with 84,223 bp) and a small single-copy region (SSC with 17,817 bp) separated by a pair of inverted repeats (IR) with a length of 25,079 bp. It was annotated with 127 genes, including 85 protein-coding genes, 34 tRNA genes, and eight rRNA genes. The 85 protein-coding genes contain photosynthesis subunit, RNA polymerase, ribosome components, maturase, protease, membrane transporter, and five proteins with unknown functions. The sizes of tRNA were from 77 to 88 bp. The lengths of rRNA were 103 bp (rrn4.5), 121 bp (rrn5), 1490 bp (rrn16), and 2809 bp (rrn23).

To investigate the evolutionary relationship of *N. peltata*, the phylogenetic analyses were performed based on chloroplast nucleotide sequences of six *Nymphoides* species, and four *Nymphaea* species (family *Nymphaeaceae*) were selected as the outer groups. The result supported that *N. peltata* has the closest phylogenetic relationship with *Nymphoides hydrophylla* (Figure 1). Moreover, the chloroplast DNA sequences of five available *Nymphoides* species formed a cluster. Consistent with previous results (Ho and Ornduff 1995), our findings indicated that *Nymphoides* formed a monophyletic clade in aquatic weeds. The complete chloroplast genome of *N. peltata* provides valuable genetic information for improving our understanding of the evolution of aquatic weeds, exploring genetic variations, and designing conservation strategies.

**Figure 1.**
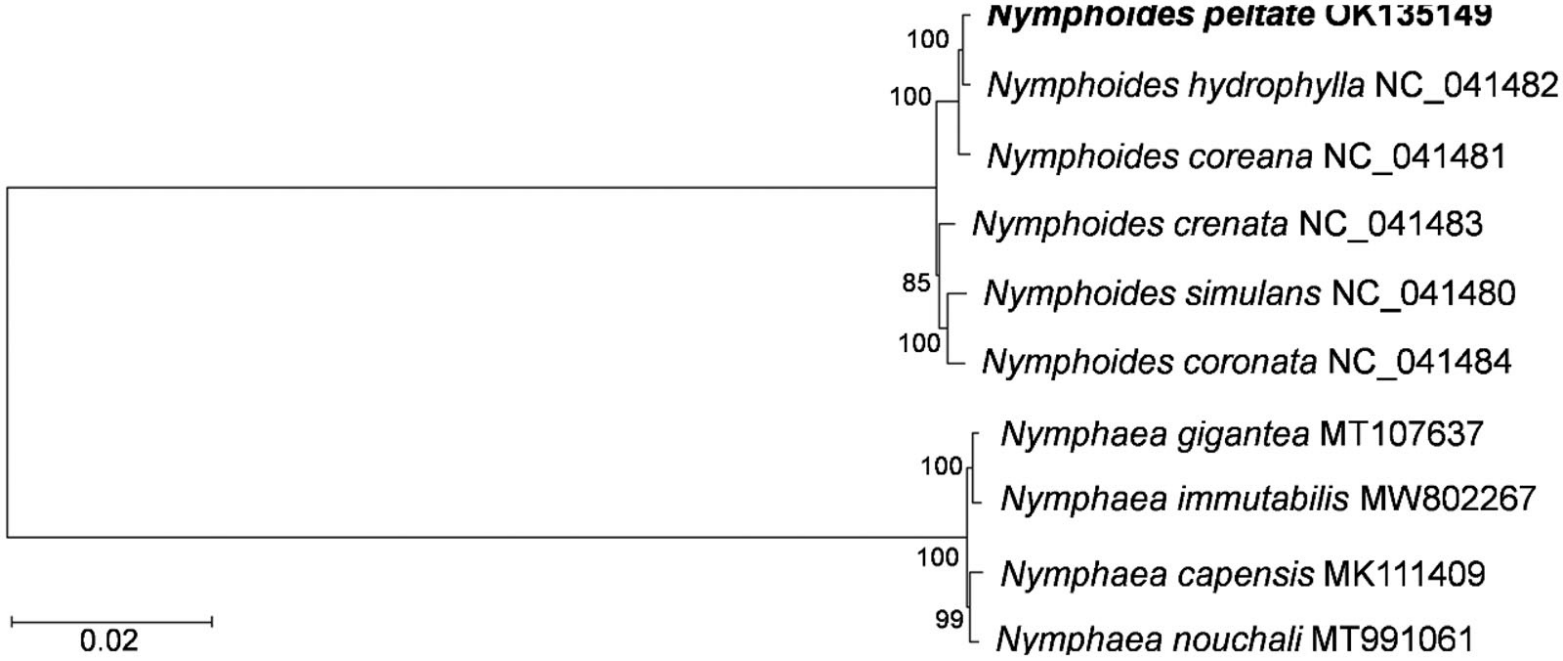
Phylogenetic tree for *Nymphoides peltata* based on the complete chloroplast genome sequences.

## Author contributions

WW designed the research. DW, ZW, and YZ collected the specimens. XZ, YL, and JZ performed the experiments and analyzed the data. All authors approved its final version.

## Data Availability

The genome sequence data that support the findings of this study are openly available in GenBank of NCBI at https://www.ncbi.nlm.nih.gov/nuccore/OK135149.1/ under the accession no. OK135149. The associated BioProject, Bio-Sample, and SRA are PRJEB38536, SAMEA6863524, and ERS4591101, respectively.
